# Risks and benefits of continuation and discontinuation of aspirin in elective craniotomies: a systematic review and pooled-analysis

**DOI:** 10.1007/s00701-022-05416-2

**Published:** 2022-11-15

**Authors:** Jonathan Rychen, Attill Saemann, Tamara Fingerlin, Raphael Guzman, Luigi Mariani, Ladina Greuter, Jehuda Soleman

**Affiliations:** 1 Department of Neurosurgery, University Hospital of Basel, Spitalstrasse 21, CH - 4031 Basel, Switzerland; 2 Faculty of Medicine, University of Basel, Basel, Switzerland; 3 Department of Clinical Studies, University Hospital of Basel, Basel, Switzerland

**Keywords:** Aspirin, Craniotomy, Hemorrhagic complication, Systematic review, Thromboembolic complication

## Abstract

**Background/aim:**

Discontinuation of aspirin (ASA) prior to elective craniotomies is common practice. However, patients treated with ASA for secondary prevention bear a higher risk for thromboembolic complications. Aim of this systematic review is to investigate the risks and benefits of perioperative continuation and discontinuation of ASA in elective craniotomies.

**Methods:**

PubMed and Embase databases were searched. Inclusion criteria were retro- and prospective studies, reporting hemorrhagic and thromboembolic complications in patients in whom ASA was either continued or discontinued perioperatively in elective craniotomies. We excluded shunt operations and emergency cases. The MINORS (Methodological index for non-randomized studies) score was used to quantify the methodological quality of the eligible studies.

**Results:**

Out of 523 publications, 7 met the eligibility criteria (cumulative cohort of 646 patients). The mean MINORS score for the comparative studies was 18.7/24 (± SD 2.07, range: 17–22) and 9/16 for the unique non-comparative study, indicating an overall weak methodological quality of the included studies. 57.1% of the patients underwent craniotomy for intra- and extra-axial tumor removal, 39.0% for bypass surgery and 3.9% for neurovascular lesions (other than bypass). In 31.0% of the cases, ASA was prescribed for primary and in 69.0% for secondary prevention. ASA was continued perioperatively in 61.8% and discontinued in 38.2% of the cases. The hemorrhagic complication rate was 3% (95% CI [0.01–0.05]) in the ASA continuation group (Con-Group) and 3% (95% CI [0.01–0.09]) in the discontinuation group (Disc-Group) (*p* = 0.9). The rate of thromboembolic events in the Con-Group was 3% (95% CI [0.01–0.06]) in comparison to 6% (95% CI [0.02–0.14]) in the Disc-Group (*p* = 0.1).

**Conclusion:**

Perioperative continuation of ASA in elective craniotomies does not seem to be associated with an increased hemorrhagic risk. The potential beneficial effect of ASA continuation on thromboembolic events needs to be further investigated in patients under ASA for secondary prevention.

**Supplementary information:**

The online version contains supplementary material available at 10.1007/s00701-022-05416-2.

## Introduction

Low-dose aspirin (acetylsalicylic acid, ASA) is an antithrombotic medication, mainly prescribed for secondary prevention of cardiovascular diseases. ASA irreversibly blocks the platelet cyclo-oxygenase enzyme system, preventing formation of thromboxane A2 and inhibiting platelet aggregation for their lifetime of approximately 10 days [1]. Discontinuation of ASA prior to elective cranial neurosurgical procedures is common practice among neurosurgeons, due to the feared potential hemorrhagic complications [13, 14, 21, 28]. A survey among neurosurgeons in Germany showed that the mean time of preoperative aspirin discontinuation is 7.3 days (range 0–21 days) [13]. On the other hand, discontinuation of aspirin bears the risk of thromboembolic complications in patients who are taking it for secondary prevention of cardio- or cerebrovascular disease [2, 5, 20, 24]. Recent publications suggested that perioperative continuation of ASA is not associated with an increased risk of hemorrhagic complications [9, 19, 23]. However, the current paucity of good evidence on this very relevant topic leads to heterogenous management strategies among neurosurgeons [13, 14]. A recent international survey stated that nearly all responders agreed that more evidence is needed concerning antithrombotic management in neurosurgery [14]. The aim of this systematic review and meta-analysis is to analyze the risk and benefits of ASA continuation and discontinuation in elective craniotomies, and consequently to provide treatment recommendations by increasing the level of evidence.

## Methods

### Literature search and inclusion criteria

The present systematic literature review was conducted according to the PRISMA guidelines [17] and adhered to a population, intervention, comparison, and outcome (PICO) protocol [25]. The intervention considered was the perioperative continuation or discontinuation of ASA in elective craniotomies. The primary outcome was the perioperative rate of hemorrhagic complications, the secondary outcome was the rate of thromboembolic complications. Full text publications of either retro- or prospective nature and written in English were included. Case reports and reviews were excluded from this analysis. Due to the fact that emergency cases are inherently of different nature than their elective counterparts, involving a much higher risk of re-bleeding, and consisting of mostly hemorrhagic conditions with subsequent different perioperative ASA management strategies, we chose to exclude emergency craniotomies and burr hole surgeries from this study. Furthermore, we excluded shunt operations since these procedures cause only minimal brain exposure/transgression. The PubMed and Embase databases were searched for relevant publications up to the end of April 2022. The used database search parameters were “(Craniotomy OR Craniotomies) AND (Aspirin OR Acetylsalicylic acid)” [4, 22].

### Qualitative analysis

For each eligible publication, the following characteristics were assessed: type of study, assessed outcome, and risk of bias. The risk of bias was evaluated as recommended in the Cochrane Handbook for Systematic Reviews of Interventions (version 5.1.0) [11], meaning that a subjective value of “high” or “low” was assigned to each publication for the risk of selection, performance, detection, attrition, and reporting bias. In addition, we assessed the MINORS (Methodological index for non-randomized studies) score [27] for each publication to quantify the methodological quality of the eligible studies. The global ideal MINORS score being 16 for non-comparative studies and 24 for comparative studies.

### Quantitative analysis

For each study, the type of surgery and the treated pathology were extracted. The perioperative ASA management was analyzed and divided in a continuation (Con-Group) and discontinuation group (Disc-Group). The duration of ASA discontinuation as well as the indication for ASA were reported. For each group, the rates of perioperative hemorrhagic and thromboembolic complications were analyzed and compared. As not all of the included studies reported thromboembolic outcomes, the pooled outcome rates for thromboembolic complications were calculated as percentage of cases with reported data. The type of hemorrhagic and thromboembolic complications was also reported.

Literature search, data extraction, qualitative and quantitative analysis were performed independently by two of the authors (J.R. and T.F.). Inconsistencies were cross-checked by the last author (J.S.).

### Statistical analysis

Outcomes are provided as percentage of cases with reported data for each outcome parameter. The risk proportion for all outcome parameters was assessed. We used risk ratio (RR) as an effect measure for our pooled outcome analysis for all studies with a reported comparison. Forest plots were calculated as sensitivity analysis only, due to the high heterogeneity and low quality of the included studies, and are therefore presented in the supplementary material. Outcome rates were compared between the two different groups using a chi-square or Fisher’s exact test. Risk and odds ratios (RR and OR) were calculated. The analyses were performed with “R statistical software” (R Statistical Software, Vienna, Austria, Version 4.0.3), running the “dmetar package” [15].

## Results

### Study identification and selection

**Fig. 1:**
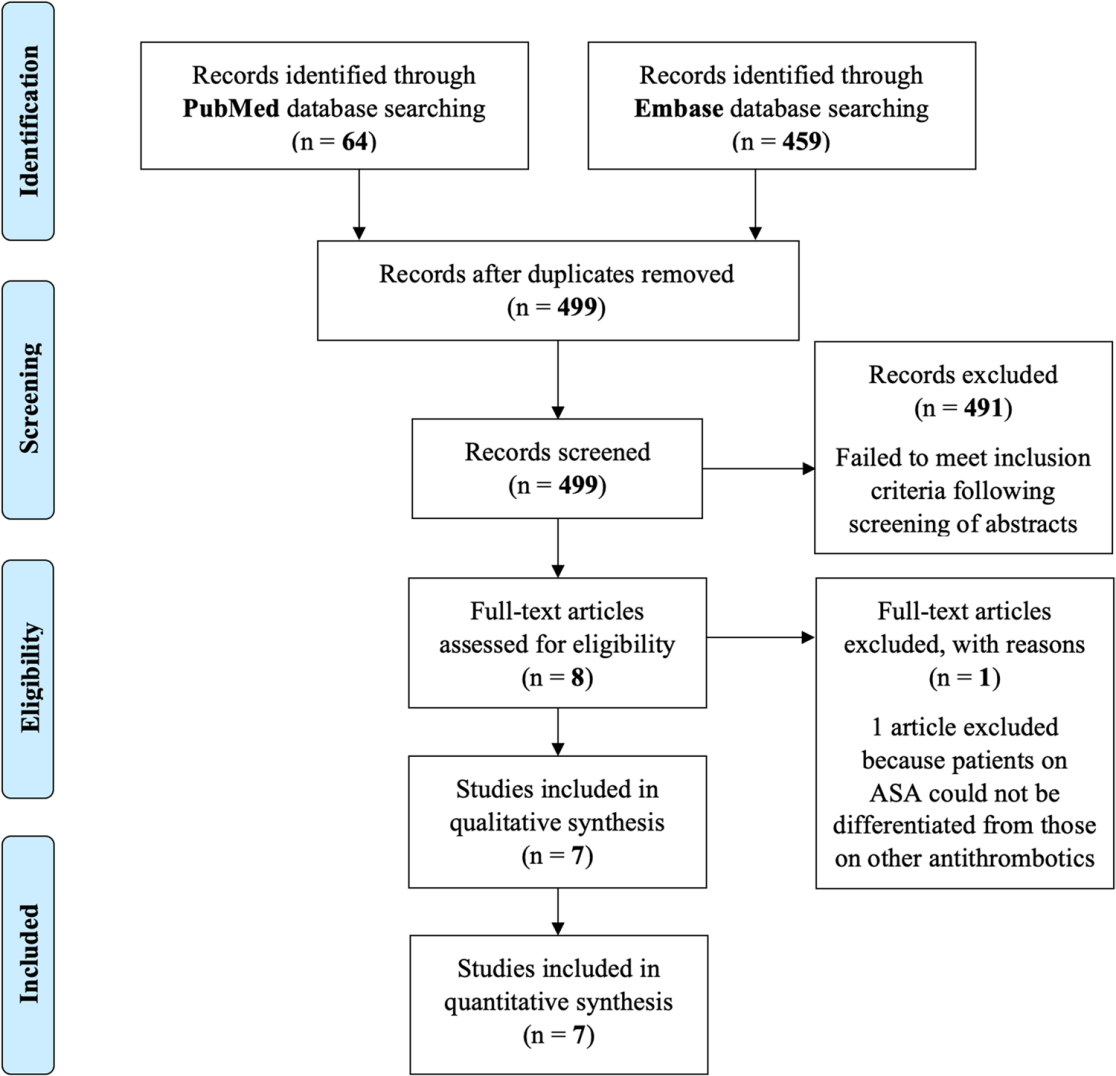
PRISMA Flow diagram. The number of records identified through database searching (on April 30, 2022), screened and assessed for eligibility, and included in qualitative and quantitative synthesis are indicated. The number of excluded records at each stage is also provided with reason for exclusion

The literature search yielded 64 publications in the PubMed and 459 publications in the Embase database. After removing 24 duplicates, the titles/abstracts of the remaining 499 publications were screened. Of these, 491 failed to meet the inclusion criteria and were therefore excluded. The remaining 8 articles were assessed for eligibility. Of these, one article was excluded because the outcome of patients on ASA could not be differentiated from those treated with other antithrombotics [18]. A total of 7 publications were included for qualitative and quantitative analysis [3, 8, 9, 12, 23, 26, 29]. The study identification and selection process are summarized in Fig. 1 [17].

### Qualitative analysis

Five of the included publications (71.4%) were retrospective cohort studies, whereas the remaining 2 (28.6%) were prospective cohort studies (Table 1). The few included prospective studies had partly different primary outcomes than those of interest of this meta-analysis, reflecting the high heterogeneity of the included studies. The mean follow-up time of the included studies was 269 days (range: 30–605 days). In most studies, a high risk for selection, performance, detection, attrition, and reporting bias was detected (Table 1). The mean MINORS score for the comparative studies was 18.7/24 (± SD 2.07, range: 17–22) and 9/16 for the unique non-comparative study, indicating an overall weak methodological quality of the included studies (Table 1).

**Table 1:** Qualitative analysis of the included studies

Publication	Year	Type of study	Outcome assessed	Mean follow-up time (days)	Risk of bias				MINORS score*
					Selection bias	Performance and detection bias	Attrition bias	Reporting bias	
Hanalioglu et al. [9]	2019	Retrospective cohort study	Hemorrhagic and thromboembolic complications	30	High	Low	Low	High	19/24
Rahman et al. [23]	2015	Retrospective cohort study	Hemorrhagic and thromboembolic complications	NR	High	High	High	High	17/24
Schubert et al. [26]	2014	Retrospective cohort study	Bypass patency and hemorrhagic complications	NR	High	High	High	Low	9/16
Grubb et al. [8]	2013	Prospective randomized	Postoperative stroke and other thromboembolic/hemorrhagic complications	605	High	High	High	High	22/24
Jussen et al. [12]	2013	Prospective randomized	Bypass patency, hemorrhagic complications and aspirin resistance	180	High	High	Low	High	20/24
Ebel et al. [3]	2021	Retrospective cohort study	Hemorrhagic and thromboembolic complications	499	High	High	High	High	17/24
Ullmann et al. [29]	2021	Retrospective cohort study	Hemorrhagic and thromboembolic complications	30	High	High	High	High	17/24

^*^MINORS: Methodological index for non-randomized studies (the global ideal score being 16 for non-comparative studies and 24 for comparative studies)

### Quantitative analysis

The 7 included publications correspond to a cumulative cohort of 646 patients. ASA was continued perioperatively in 399 patients (61.8%) and discontinued in 247 patients (38.2%). The duration of perioperative ASA discontinuation is provided in Tables 2 and 3.

**Table 2:** Quantitative analysis of hemorrhagic complications

Publication	Perioperative management of ASA	Number of patients	Type of surgery	Site of surgery	Rate of hemorrhagic complications (95% CI)	*P* value°
Hanalioglu et al. [9]	Discontinuation (> 7 days preop)	104	Craniotomy for tumor surgery	71.3% supratentorial / 28.7% infratentorial 50% intra-axial/50% extra-axial	0.9% (0.0–5.1)	1
	Continuation	119		79.4% supratentorial/20.6% infratentorial 58.7% intra-axial/41.3% extra-axial	0.8% (0.0–4.3)	
Rahman et al. [23]	Discontinuation (Days not specified)	55	Craniotomy for tumor surgery	NA	7.3% (0.4–14.1)	1
	Continuation	28			7.1% (0.0–16.7)	
Schubert et al. [26]	Continuation	103	Craniotomy for bypass	100% supratentorial	1.9% (0.0–4.6)	-
Grubb et al. [8]	Continuation	93	Craniotomy for bypass	100% supratentorial	3.2% (0.0–6.8)	
Jussen et al. [12]	Continuation	56	Craniotomy for bypass	100% supratentorial	0%	
Ebel et al. [3]	Discontinuation (Mean: 13 days preop)	25	Craniotomy for neurovascular lesions	96% supratentorial/4% infratentorial	4% (0–12)	
Ullmann et al. [29]	Discontinuation (≤ 5 days preop: 19 patients) (> 5 days preop: 44 patients)	63	Craniotomy for tumor surgery	90.5% supratentorial/9.5% infratentorial 55.6% intra-axial/44.4% extra-axial	0%	

*Con-Group* continuation group, *Disc-Group* discontinuation group, *NA* not available

° All comparisons computed with Fisher’s exact test

**Table 3:** Quantitative analysis of thromboembolic complications

Publication	Perioperative management of ASA	Number of patients	Type of surgery	Site of surgery	Rate of thromboembolic complications (95% CI)	*P* value°
Hanalioglu et al. [9]	Discontinuation (> 7 days preop)	104	Craniotomy for tumor surgery	71.3% supratentorial / 28.7% infratentorial 50% intra-axial/50% extra-axial	1.9% (0.2–6.5)	0.60
	Continuation	119		79.4% supratentorial / 20.6% infratentorial 58.7% intra-axial/41.3% extra-axial	0.8% (0.0–4.3)	
Rahman et al. [23]	Discontinuation (Days not specified)	55	Craniotomy for tumor surgery	NA	1.8% (0.0–5.3)	1
	Continuation	28			3.6% (0.0–10.4)	
Grubb et al. [8]	Continuation	93	Craniotomy for bypass	100% supratentorial	3.2% (0.0–6.8)	-
Ebel et al. [3]	Discontinuation (Mean: 13 days preop)	25	Craniotomy for neurovascular lesions	96% supratentorial/4% infratentorial	12% (0.0–24.7)	
Ullmann et al. [29]	Discontinuation (≤ 5 days preop: 19 patients) (> 5 days preop: 44 patients)	63	Craniotomy for tumor surgery	90.5% supratentorial/9.5% infratentorial 55.6% intra-axial/44.4% extra-axial	11.1% (3.1–19.0)	

*Con-Group* continuation group, *Disc-Group* discontinuation group, *NA* not available

° Comparison computed with Fisher’s exact test

57.1% of the patients underwent craniotomy for intra- and extra-axial tumor removal (*n* = 369), 39.0% for bypass surgery (*n* = 252), and 3.9% for neurovascular lesions (other than bypass) (*n* = 25). More detailed information about the type and site of surgeries is outlined in Tables 2 and 3.

#### Hemorrhagic complications

All of the studies reported the hemorrhagic complication rate. 8 out of 399 patients in the Con-Group and 6 out of 247 patients in the Disc-Group suffered a hemorrhagic event. This corresponds to an overall pooled hemorrhagic complication rate of 3% (95% CI [0.01; 0.05]) in the Con-Group and 3% (95% CI [0.01; 0.09]) in the Disc-Group (*x*^2^ = 0.006, RR 0.83, 95% CI [0.29; 2.35] *p* = 0.9 | OR 0.82, 95% CI [0.28; 2.5]) (Table 2; Suppl. Figs. 1–2).

Of 14 patients with hemorrhagic complications, the type of hemorrhage was reported for 7 cases (50.0%). Four patients (57.1%) experienced a postoperative subdural hematoma, one patient (14.3%) suffered from an intracerebral hematoma, one further patient (14.3%) had a subarachnoid hemorrhage, and one patient (14.3%) a subgaleal hematoma.

#### Thromboembolic complications

Only five studies reported the thromboembolic complication rate. 5 out of 240 patients in the Con-Group and 13 out of 247 patients in the Disc-Group suffered a thromboembolic event. This corresponds to an overall pooled thromboembolic complication rate of 3% (95% CI [0.01; 0.06]) in the Con-Group compared to 6% (95% CI [0.02; 0.14]) in the Disc-Group (*x*^2^ = 2.6, RR 0.39, 95% CI [0.14–1.09], *p* = 0.1 | OR 0.39 95% CI [0.13–1.13]) (Table 3; Suppl. Figs. 3–4).

In all the 18 patients with thromboembolic complications, the type of complication was reported. Nine patients (50.0%) suffered from a stroke, four patients (22.2%) from a deep vein thrombosis, three patients (16.7%) from a myocardial infarction, and two patients (11.1%) from a pulmonary embolism.

#### Indication for ASA

The indication for ASA was reported in 558 cases (86.4%). In 173 cases (31.0%), ASA was prescribed for primary prevention, whereas 385 patients (69.0%) had ASA for secondary prevention. In the Con-Group, 75 patients (18.8%) had ASA for primary, whereas 324 patients (81.2%) for secondary prevention. In the Disc-Group, 98 patients (61.6%) had ASA for primary, whereas 61 patients (38.4%) for secondary prevention. In summary, the Con-Group had significantly more patients with ASA for secondary prevention (81.2%) than the Disc-Group (38.4%) (*p* < 0.001).

#### Subgroup analysis without bypass surgeries

Because of the high proportion (39%) of bypass surgery in our review, which doesn’t represent the reality of daily neurosurgical practice, we performed a subgroup analysis of the primary and secondary outcomes excluding bypass surgeries. In this subgroup analysis, no difference between the Disc- and Con-Group was found for hemorrhagic complication rates (3% (95% CI 0.0–0.2) in the Con-Group and 3% (95% CI 0.01–0.09) in the Disc-Group (OR 1.2 95% CI [0.25–7.49], *p* = 1)), while the rate of thromboembolic complications was lower in the Con-Group (2% (95% CI 0.0–0.07)) compared to the Disc-Group (6% (95% CI 0.02–0.14), OR 4.01, 95% CI [0.9–37.1], *p* = 0.06), however, lacked statistical significance (Suppl. Figs. 5–8).

## Discussion

Based on our systematic review of the literature, perioperative continuation of ASA in elective craniotomies does not seem to be associated with a higher risk of hemorrhagic complications. Concerning thromboembolic events, our pooled analysis shows a trend towards a beneficial effect of ASA continuation; however, the results didn’t reach statistical significance. The qualitative analysis revealed a relatively low mean MINORS score [27] (18.7/24 for the comparative studies and 9/16 for the non-comparative study), reflecting an overall weak methodological quality of the included studies. Hence, the findings presented in this study have to be interpreted with caution. The lack of high-quality studies on this very important topic is probably due to the fear of many neurosurgeons to continue ASA perioperatively and potentially experience hemorrhagic complications. This review aimed to reduce this apprehension and encourage neurosurgeons to produce better evidence on this topic.

The question whether the duration of preoperative ASA discontinuation influences the rate of hemorrhagic complication was addressed by two studies in this review. Neither Ebel et al. nor Ullmann et al. found a significant difference in hemorrhagic complications with shorter (< 5 days) or longer (> 5 days) ASA discontinuation time [3, 29]. The publication by Rahman et al. was the only one comparing the need for surgical revision after a hemorrhagic complication. They didn’t find a statistically significant difference between the ASA Con and Disc-Group (7.1% and 3.7% respectively; *p* = 1). Likewise, the authors didn’t find a difference in mortality rates (0% in the ASA Con-Group and 3.7% in the Disc-Group; *p* = 0.6) and also no difference in functional outcomes [23]. It is interesting to mention that the study by Ebel et al. was the one with the highest thromboembolic complication rate (12%) in patients who underwent cerebrovascular surgery and discontinued ASA. This might be due to the surgical manipulation of the vasculature and/or due to ASA discontinuation. Continuation of ASA could potentially provide protection from thromboembolic complications, particularly in patients with atherosclerotic vasculature, and especially when undergoing cerebrovascular surgery.

Almost 40% of the included patients in this systematic review underwent bypass surgery. This is due to the fact that this procedure is typically performed under ASA therapy to prevent bypass thrombosis/failure [8, 12]. However, such a high proportion of bypass surgeries does not represent the reality of daily neurosurgical practice. In the subgroup analysis without revascularization procedures, the hemorrhagic and thromboembolic complication rates were similar to those of the overall cohort, making our results valid for daily neurosurgical practice.

Overall, the perioperative management of ASA appears very heterogenous among neurosurgeons [13, 14]. Our group performed an international survey of practice on the topic of antithrombotic management of patients undergoing elective craniotomy [14]. Nearly all responders (93%) agreed that more evidence is needed concerning antithrombotic management in neurosurgery. Around half of the responders considered it safe to continue or resume ASA within 3 days for bypass (55%) or vascular (49%) surgery, whereas significantly less responders considered it safe for tumor craniotomies (26%) and skull base procedures (14%) [14].

Concerning aneurysm surgery, we expect in the future an increasing number of patients under ASA medication due to the growing evidence of the protective effect of ASA against aneurysm growth and rupture[10, 30, 31]. Considering this aspect, the results of this study are even more relevant. The largest included study in this systematic review is that of Hanalioglu et al.[9]. They analyzed the effect of perioperative ASA continuation in tumor surgeries (intra- and extraaxial tumors) and didn’t find an increased rate of hemorrhagic complications. Although without a significant difference, they found a slightly higher rate of thromboembolic complications in the ASA Disc-Group. Skull base tumors were found to be independent predictors of thromboembolic complications. With the results of this systematic review and pooled analysis, we see a clear trend toward the protective effect of ASA continuation on thromboembolic complications. Regarding transsphenoidal pituitary surgery and continuation of ASA therapy, the current literature is very scarce. Indeed, we could find only one publication, reporting no hemorrhagic complication in a small cohort of 9 patients who underwent transsphenoidal surgery under ASA therapy [19].

To distinguish the different indications for ASA is essential. In the past, ASA was prescribed for secondary and primary prevention of cardio- and cerebrovascular diseases[1]. However, recent studies have shown that ASA should no longer be recommended for primary prevention [6, 7, 16]. In our study, ASA was prescribed mostly for secondary prevention. Even though one third of the patients were taking it for primary prevention, the study’s conclusions are still valuable. The Con-Group had significantly more patients with ASA for secondary prevention than the Disc-Group. In other words, the burden of cardiovascular diseases was lower in the Disc-Group. If solely patients with ASA for secondary prevention had been included in this study, the beneficial impact of ASA continuation on thromboembolic events might have been higher and might have reached statistical significance. Based on our findings and the lack of higher evidence, it seems reasonable and pragmatic to recommend perioperative ASA continuation for patients taking it for secondary prevention, since the risk of thromboembolic complications is higher for these patients. It seems that continuing ASA is at least non-inferior to discontinuing ASA in terms of hemorrhagic complications.

### Strengths

To the best of our knowledge, this study is the first analysis reporting risks and benefits of perioperative continuation of ASA in elective craniotomies. It addresses a very relevant topic of daily neurosurgical practice, for which the paucity of evidence leads to partly arbitrary management strategies. Our pooled analysis provides some evidence for the safety of ASA continuation in elective cranial procedures. We recommend further investigations with an adequate powered randomized controlled trial (RCT) to confirm the beneficial trend of ASA continuation on thromboembolic events.

### Limitations

The majority of the included studies are of retrospective nature and comprise small patient cohorts. Moreover, the primary outcomes of the included prospective studies partly differ from the primary outcome of this analysis, reflecting the high heterogeneity of the included studies. These important limitations lead to a weak statistical validity. However, we believe that it is still important to share such a pooled analysis with the neurosurgical community, even if the quality of the included studies is low, in order to provide other research groups with the best available data to estimate appropriate sample sizes for future RCTs. Therefore, the forest plots representing the results of a meta-analysis calculation are presented but solely as supplementary material, since these calculations could be random at best due to the low quality of studies included. The calculated MINORS score indicates an overall weak methodological quality of the included studies along with a relatively high risk for selection, performance, detection, attrition, and reporting bias. We disclose that the studies by Ebel et al. and Ullmann et al. are studies performed by our own research group [3, 29]. Even though the literature search was performed systematically, we cannot exclude a certain selection bias in this regard. Only two databases (PubMed and Embase) and solely articles in English were searched, which carries a risk of omitting data. Finally, we can’t exclude a general publication bias due to unpublished negative results, which could not be included in this systematic review.

## Conclusion

Based on these results, the perioperative continuation of ASA in elective craniotomies does not seem to be associated with a higher hemorrhagic risk. The potential beneficial effect of ASA continuation on thromboembolic events in patients taking it for secondary prevention needs to be further investigated in well-designed randomized controlled trials.

## Supplementary information

Below is the link to the electronic supplementary material.

Supplementary file1 (DOCX 783 KB)

## Abbreviations

ASA: Acetylsalicylic acid
CI: Confidence interval
Con-Group: Continuation group
Disc-Group: Discontinuation group
RCT: Randomized controlled trial

## Declarations

### Previous presentation

Part of this study was presented as an oral presentation at the annual meeting of the Swiss Society of Neurosurgery (16/09/21- 17/09/21; Virtual meeting).
