# Supplementary material for: Risks and benefits of continuation and discontinuation of aspirin in elective craniotomies: a systematic review and pooled-analysis
Source: Acta Neurochir (Wien). 2022 Nov 15;165(1):39–47. doi: 10.1007/s00701-022-05416-2 (PMC9840583; doi:10.1007/s00701-022-05416-2)
Supplement: Supplementary file 1 — Supplementary file1 (DOCX 783 KB) [file 701_2022_5416_MOESM1_ESM.docx]

**Supplementary Figure 1: Single-arm meta-analysis of overall pooled outcome rate of hemorrhagic complications in the ASA Con-Group**

**
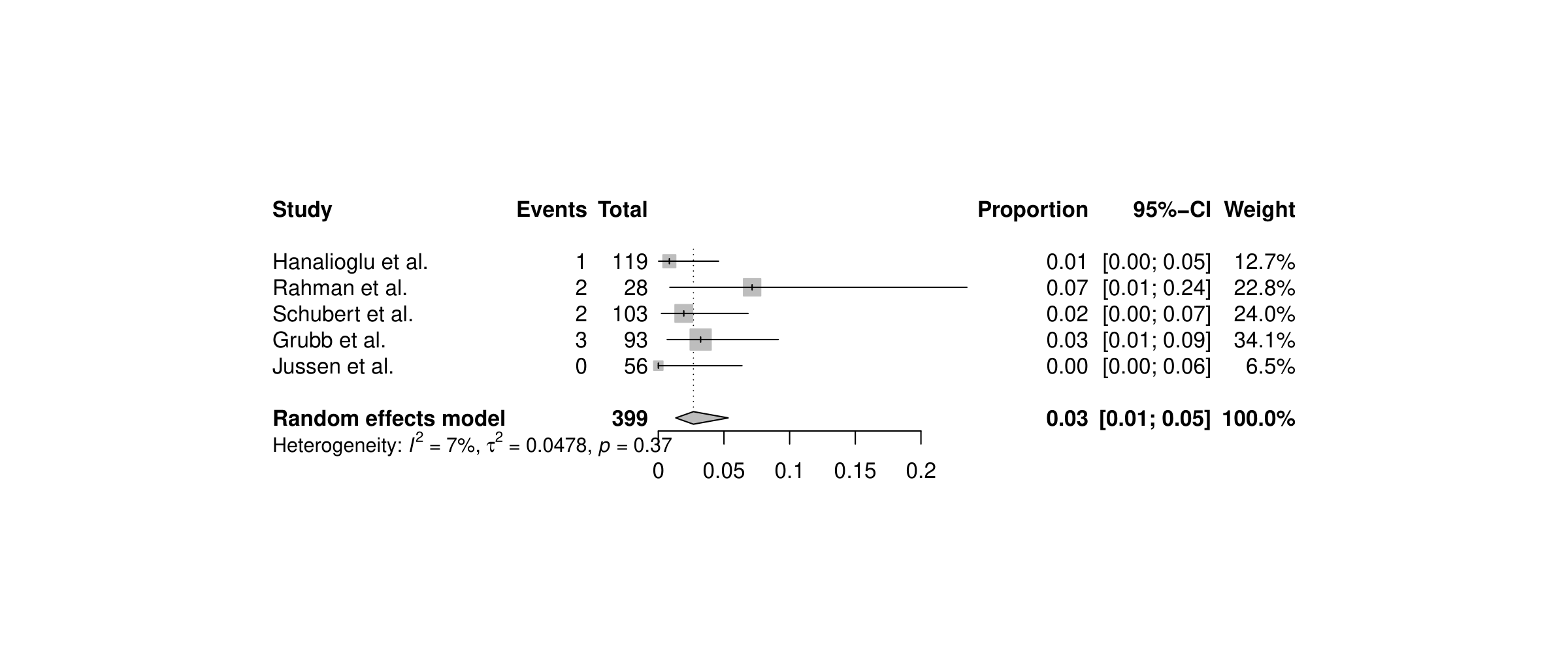
Supplementary Figure 2: Single-arm meta-analysis of overall pooled outcome rate of hemorrhagic complications in the ASA Disc-Group**

**
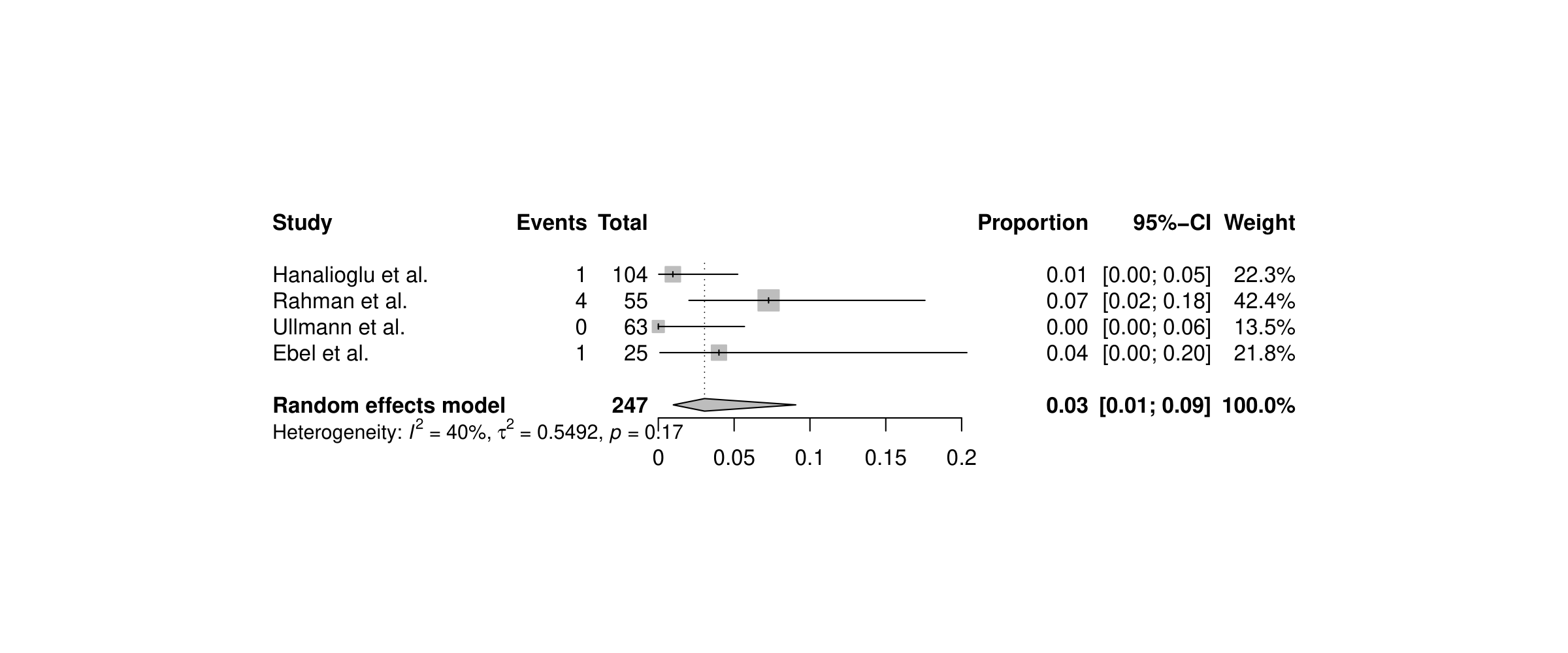
Supplementary Figure 3: Single-arm meta-analysis of overall pooled outcome rate of thromboembolic complications in the ASA Con-Group**

**
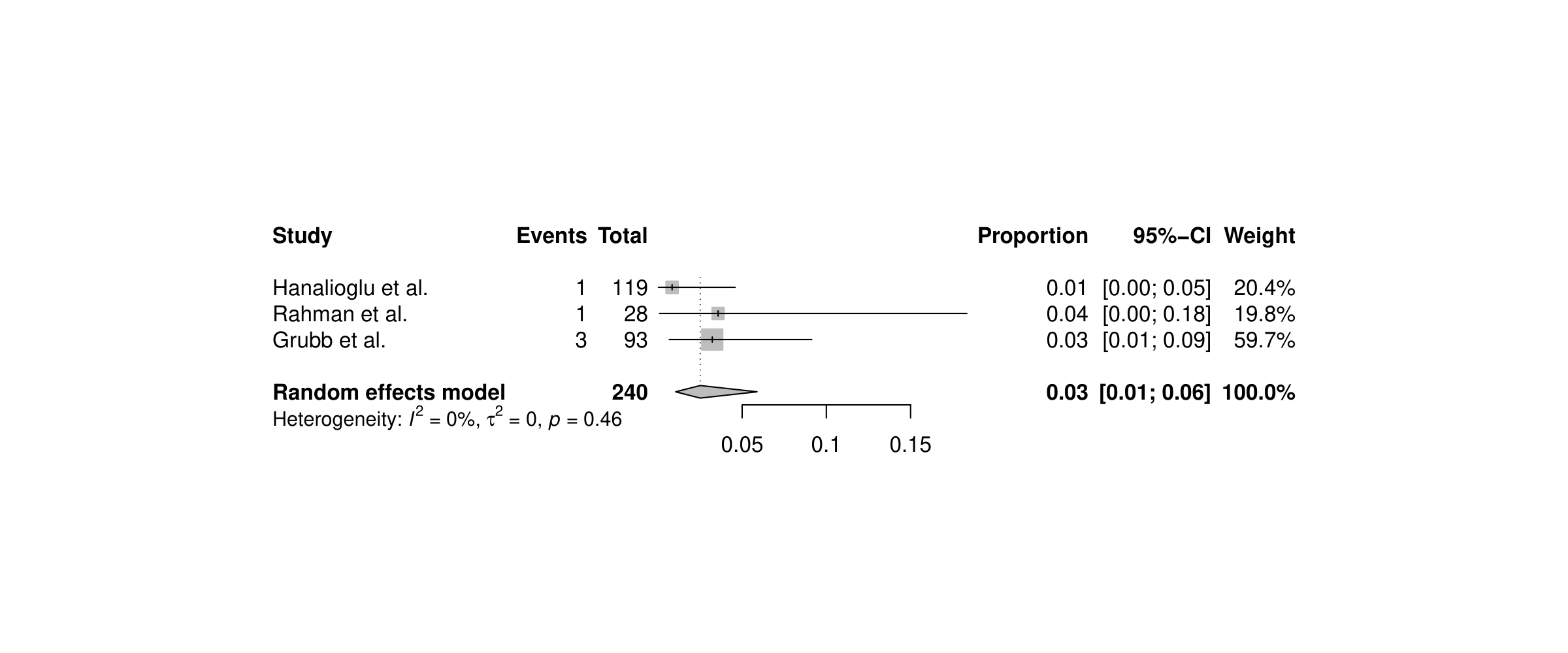
**

**Supplementary Figure 4: Single-arm meta-analysis of overall pooled outcome rate of thromboembolic complications in the ASA Disc-Group**

**
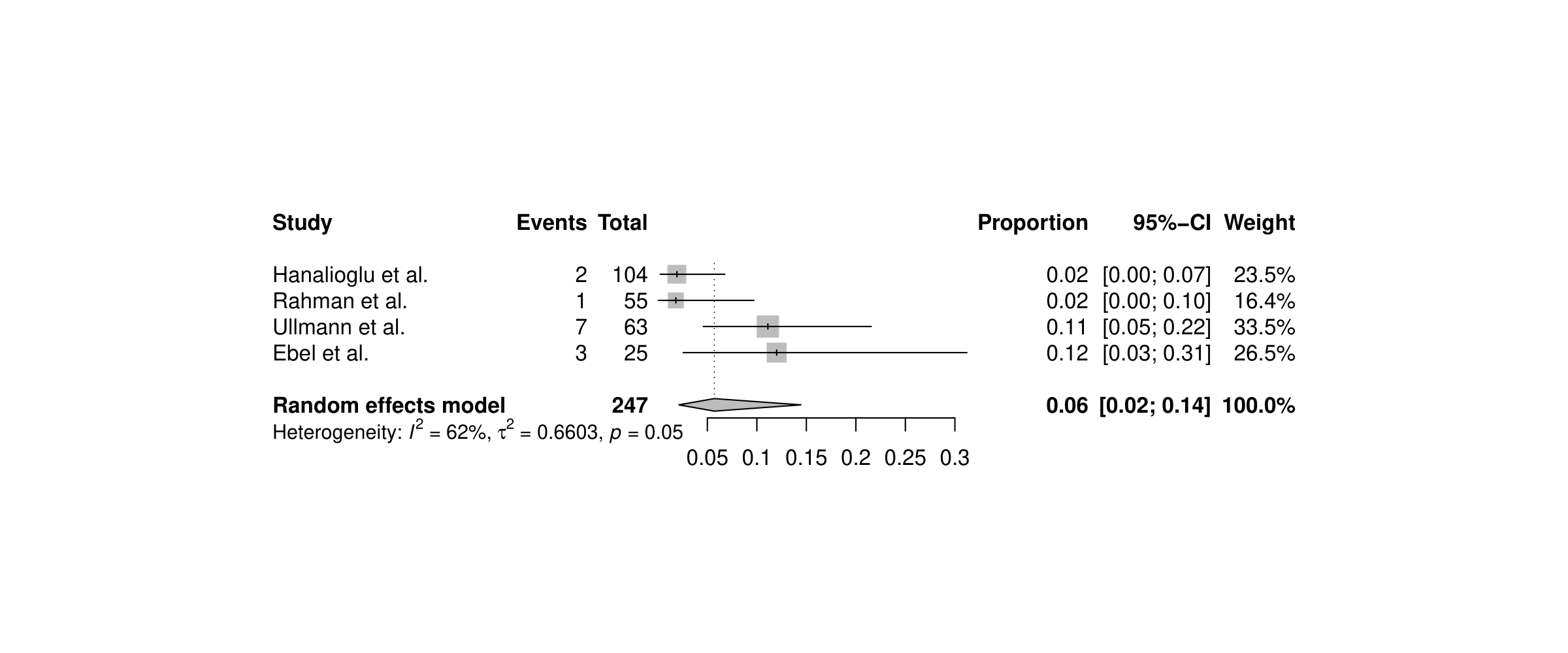
Supplementary Figure 5: Single-arm meta-analysis of overall pooled outcome rate of hemorrhagic complications in the ASA Con-Group in the subgroup without bypass surgeries**

**Supplementary Figure 6: Single-arm meta-analysis of overall pooled outcome rate of hemorrhagic complications in the ASA Disc-Group in the subgroup without bypass surgeries**

**Supplementary Figure 7: Single-arm meta-analysis of overall pooled outcome rate of thromboembolic complications in the ASA Con-Group in the subgroup without bypass surgeries**

**Supplementary Figure 8: Single-arm meta-analysis of overall pooled outcome rate of thromboembolic complications in the ASA Disc-Group in the subgroup without bypass surgeries**

Abbreviations:

ASA: Aspirin; Con-Group: Continuation Group; Disc-Group: Discontinuation Group
